# Resilience Programs for Children and Adolescents: A Systematic Review and Meta-Analysis

**DOI:** 10.3389/fpsyg.2021.754115

**Published:** 2021-11-22

**Authors:** Tatiana Matheus Pinto, Paulo Guirro Laurence, Cristiane Rufino Macedo, Elizeu Coutinho Macedo

**Affiliations:** ^1^Social and Cognitive Neuroscience Laboratory and Developmental Disorders Program, Center for Health and Biological Sciences, Mackenzie Presbyterian University, São Paulo, Brazil; ^2^Brazilian Cochrane Center, Escola Paulista de Medicina, Universidade Federal de São Paulo, São Paulo, Brazil

**Keywords:** resilience, program, children, adolescents, systematic review

## Abstract

Resilience may be defined as the ability to recover and adapt to adverse situations. Given that resilience involves cognitive and behavioral aspects, it could be promoted based on strategies that favor them, especially during childhood and adolescence. As a result, several resilience-focused programs have been developed and studied. This systematic review of Randomized Controlled Trials (RCTs) aimed to assess resilience-focused programs for children (<12 years old) and adolescents (12–22 years old) compared to active (treatment as usual, other program modalities, and educational curriculum at school) or inactive (waiting list, no treatment) control groups. We performed a systematic review of meta-analyses of RCTs. The following databases were searched: Cochrane Central Register of Controlled Trials (CENTRAL), PubMed, Embase, and PsycINFO. Two authors independently selected the studies, extracted the data, and assessed the studies’ risk of bias. Meta-analyses of random effects were conducted to calculate the standard mean differences (SMD) and 95% confidence interval (CI) of program effectiveness. Of the 17 RCTs that met the inclusion criteria, 13 provided sufficient data to assess the effectiveness of the programs after their implementation. Meta-analyses indicated overall effectiveness of the programs in promoting resilience (SMD = 0.48, 95% CI [0.15, 0.81], *p* = 0.0077). The subgroup analysis indicated effectiveness only among adolescents’ resilience (SMD = 0.48, 95% CI [0.08, 0.88], *p* = 0.02). The follow-up analysis also indicated evidence of continuation of results within a period of up to 6 months up (SMD = 0.12, 95% CI [−0.44, 0.69], *p* = 0.02). These results indicated the effectiveness of promoting resilience, especially in adolescents, and its continuation in follow-up analyses. These findings are promising in the field of resilience programs; however, further studies are necessary to analyze the different possible characteristics of programs and their results.

**Clinical Trial Registration:** [https://www.crd.york.ac.uk/prospero/display_record.php?ID=CRD42020179874], [CRD42020179874].

## Introduction

In a constant changing world where people need to adapt and deal with new challenges daily, resilience is extremely important, as resilient individuals adapt better to life challenges and have higher levels of functionality and quality of life (Leppin et al., 2014). The American Psychological Association (APA) defines resilience as the ability to recover and adapt to situations of adversity, trauma, threats, or sources of stress. Being resilient, however, does not necessarily mean that individuals will not experience difficulties or discomfort when exposed to such situations [American Psychological Association (APA), 2020].

Although resilience was initially seen as a fixed trait, it is currently considered a dynamic process that can be modified throughout life (Chmitorz et al., 2018; Masten, 2018). This perspective involves cognitive, attitudinal, and behavioral aspects that can be learned. Therefore, resilience capacity can be enhanced based on strategies that develop these aspects [American Psychological Association (APA), 2020].

Given that resilience can be strengthened with strategies that favor cognitive, attitudinal, and behavioral aspects, programs focused on resilience have been developed. Laird et al. (2019) reported that such programs can be implemented preventively to reduce susceptibility to psychopathologies or as treatment for individuals who already have a mental disorder diagnosis, such as depression (Laird et al., 2019).

Despite many diverse populations may benefit from these interventions, some specific periods of development might be more conducive for implementing strategies that promote resilience. One of these periods is childhood because of the greater brain plasticity and learning capacity during this stage (Masten and Barnes, 2018). Although it is difficult to define childhood with exact ages, it may be considered up to 11 years of age (DelGiudice, 2018). Another favorable development phase for the implementation of such interventions is adolescence, which is characterized by the acquisition of executive functions, enabling greater capacity for planning and self-regulation. In addition, this stage of development is characterized by the tendency to associate with peers, which may influence an individual’s life trajectory to a more positive path if they associate with peers who exert positive influences (Masten and Barnes, 2018). Adolescence may be understood as the transition period between childhood and adulthood and considered up to 22 years of age (Goossens, 2006).

Resilience-focused interventions may involve different approaches, such as the use of pharmacology to treat diseases, physical activities and exercises, and psychological or psychotherapeutic methods. Psychotherapeutic interventions involve, among others, psychoeducational techniques, cognitive-behavioral therapy, problem-solving therapy, and mindfulness. Such psychotherapy-based resilience programs are an interesting intervention possibility as they may show a many beneficial effects, such as increasing momentary well-being, decreasing symptoms of psychopathologies, and promoting individuals’ ability to recover from adversity (Laird et al., 2019).

Several resilience-focused programs that were developed may have a wide range of characteristics. They may be aimed toward populations of different ages, be held individually or in groups, be implemented in person or remotely, have varying number and duration of sessions, and have different theoretical approaches (Dray et al., 2017; Helmreich et al., 2017). Given this diversity of characteristics, some review studies and meta-analyses have been conducted to evaluate their effectiveness (Leppin et al., 2014; Vanhove et al., 2015; Dray et al., 2017; Hodder et al., 2017; Fenwick-Smith et al., 2018).

These resilience programs have been showing effectiveness in many diverse outcomes. Studies found evidence of such programs in promoting performance and emotional well-being of adult populations in organizational contexts (Vanhove et al., 2015), reducing stress and depression, and promoting resilience in adults for up to three months after the program completion (Leppin et al., 2014). In addition, improvements in resilience and protective factors, such as coping skills, internalizing behaviors, and self-efficacy (Fenwick-Smith et al., 2018) and reduced anxiety symptoms and psychological distress (Dray et al., 2017) were found among children after attending universal resilience programs. Among adolescents, such programs seem to reduce internalizing problems (Dray et al., 2017) and even reduce the use of illicit substances (Hodder et al., 2017). Despite these promising results, few studies have analyzed the long-term results of such programs and those that did indicate a tendency of effectiveness decrease as the time passes (Vanhove et al., 2015; Dray et al., 2017).

Although the results of such programs seem promising for different outcomes, further studies are needed to analyze their different characteristics and results in other outcomes, such as resilience itself (Leppin et al., 2014; Helmreich et al., 2017; Chmitorz et al., 2018), especially among children and adolescents, given that these developmental stages are considered to be the most favorable phases for implementing strategies that promote resilience (Masten and Barnes, 2018).

Therefore, the present study aimed to answer the following question: are resilience programs with psychotherapeutic approaches for children and adolescents effective in promoting resilience?

## Objective

The aim of this study was to assess the effectiveness of resilience-focused programs in promoting resilience in children and adolescents compared to active (treatment as usual, other program modalities, educational curriculum at school) or inactive (waiting list, no treatment) control groups.

## Method

This systematic review was registered at PROSPERO (CRD42020179874). It followed the guidelines of the Preferred Reporting Items for Systematic Review and Meta-Analysis (Page et al., 2021).

### Eligibility Criteria

The criteria for inclusion in this review were randomized controlled trials (RCTs), studies with a sample of children (<12 years old) or adolescents (12 to 22 years old), studies that implemented programs with psychological/psychotherapeutic approaches (psychoeducational, based on mindfulness, cognitive-behavioral therapy, art therapy, among others, and these programs may be implemented individually or in group, face-to-face or online, and involve or not the parents), and studies that assessed resilience as an outcome.

The exclusion criteria were studies that had no control group or without randomization (wrong study design), samples of adults (wrong population), interventions that had no psychological/psychotherapeutic approach (wrong intervention), studies that did not report resilience as an outcome (wrong outcome), protocol records, and abstracts in conferences.

### Search Methods

The search strategy was developed with the assistance of the Information Specialist of Brazil Cochrane, according to the Cochrane Handbook for Systematic Reviews of Interventions, Chapter 6. The following electronic databases were searched: Cochrane Central Register of Controlled Trials (CENTRAL, Embase *via* Elsevier, PubMed, and PsycINFO. Key search terms included “child” OR “children” OR “adolescent” OR “adolescents” AND “resilience” OR “resiliency” AND “program.” The searches were not restricted by date, language, publication status, or publication format. Full detailed search strategy can be found in Appendix 1.

### Study Selection Process

The selection of studies followed the PRISMA guidelines and was conducted by two independent reviewers (TMP and PGL). First, duplicate records were removed. The titles and abstracts were screened and selected as either potentially eligible or excluded. Those selected as potentially eligible had their full text retrieved. Disagreements in the selection process were discussed with the third author (ECM), and the final inclusion decision of the studies was reached by consensus. The selection of studies was carried out using the *revtools* R package (Westgate, 2019a,b) of R software (R 3.6.3 for Windows).

### Data Extraction

After selecting the studies, two review authors (TMP and PGL) independently extracted the data in duplicate. Discrepancies were resolved by consensus. We developed data extraction forms to facilitate the standardization of data extraction. The extraction sheet contained the following elements: year of publication, country of study, sample size, age and sex of participants, name of the program, number of sessions and length per session, program implementation setting, theoretical approach, scales used to access the outcome, and time of assessment.

The articles’ risk of bias was assessed according to the Cochrane Handbook for Systematic Reviews of Interventions (Higgins and Green, 2011) and judgments were made by consensus. The following domains were assessed: selection bias (random sequence generation and allocation concealment), performance bias (blinding of participants and personnel), detection bias (blinding of outcome assessment), attrition bias (incomplete outcome data), reporting bias (selective reporting), and other biases (cross-contamination). For overall bias, we considered a low overall risk only if all domains were judged as low. We judged studies with some concerns if they were considered to be at an unclear risk for multiple domains but not to be at a high risk for any domain. We judged them as high overall bias if they were considered to be at a high risk in at least one domain. We used the *Robvis* tool to create risk of bias plots (McGuinness and Higgins, 2020).

For the meta-analysis, we retrieved the following data from each study: number of participants in the experimental and control groups and means (M) and standard deviations (SD) from before and after a program’s implementation and from follow-ups of studies that performed this assessment.

### Data Analysis

R (R Core Team, 2020) and the *meta* package (Balduzzi et al., 2019) were used to perform the meta-analysis. Authors of the papers that did not provide enough data for their inclusion in the meta-analysis were contacted. The procedures for calculations followed the recommendations of Harrer et al. (2019). We calculated the standard mean differences (SMD) of the programs’ effectiveness. We defined a 95% confidence interval (CI) and a statistically significant value of *p* < 0.05.

Subgroup analyses were performed separately with samples of children (up to 11 years old) and adolescents (12 to 22 years old). We also analyzed the short-term (≤3 months), mid-term (3–6 months), and long-term (> 6 months) follow-up results.

Heterogeneity (*I*^2^) among the studies was also assessed. This measure helps provide data on the consistency of results. This percentage was analyzed following the recommendations of Higgins et al. (2003): 25% might be considered as low heterogeneity, 50% as moderate heterogeneity, and 75% as high heterogeneity (Higgins et al., 2003; Harrer et al., 2019). Therefore, the greater the heterogeneity, the greater the differences between the results of the studies (Higgins et al., 2003).

The fact that studies reporting higher effect sizes are more likely to be published than those with lower effects may lead to publication bias. Given this, publication bias was assessed according to Hoffman’s (2019) recommendations. This data was presented through a visual analysis in a funnel plot, which considered the SMD vs. the standard error (SE) of studies. Intersections on the *x*-axis closer to zero do not indicate considerable asymmetry, which might be interpreted as a low risk of publication. Statistical significance was assessed using the Egger’s test (Hoffman, 2019).

## Results

### Search Results

**FIGURE 1:**
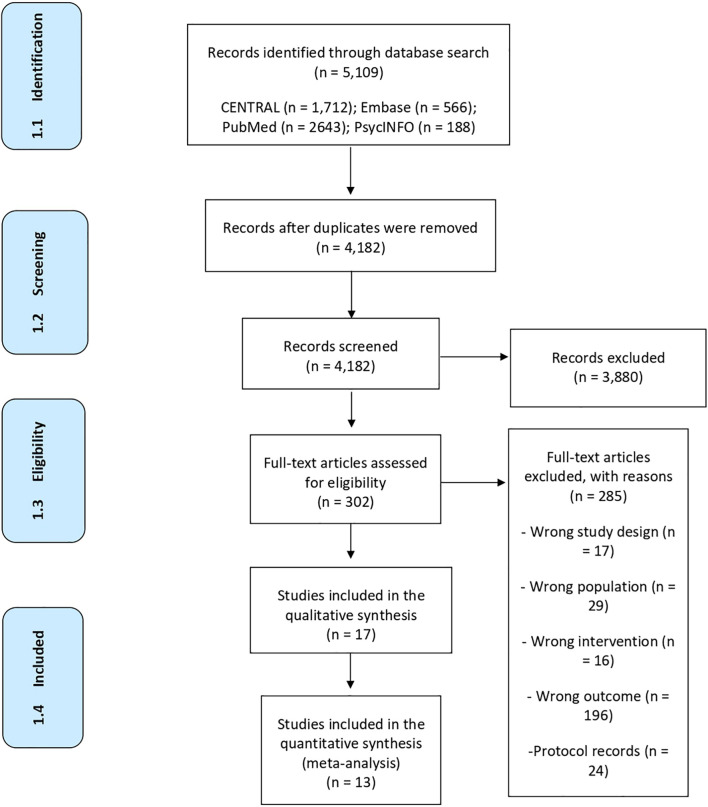
Study flow diagram.

The search resulted in 5,109 records. After duplicates were removed, 4,182 records remained. After screening the titles and abstracts, 302 studies were selected as potentially eligible and had their full text accessed for the final inclusion decision. Of the 302 studies, 285 were excluded for the following reasons: 17 had the wrong study design, 29 had the wrong population, 16 had the wrong intervention, 196 had the wrong outcome, 24 were protocol records, and 3 were abstracts in conferences. Therefore, 17 studies were included in this review. Figure 1 shows a flow diagram of the study.

### Study Characteristics

#### Year of Publication

**FIGURE 2:**
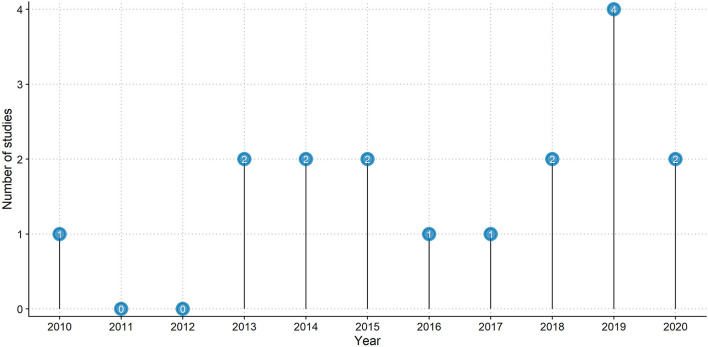
Studies published per year.

In this systematic review, we included 17 RCTs. Four studies were published in 2019 (Adibsereshki et al., 2019; Druker et al., 2019; Volanen et al., 2019; Zhang et al., 2019); two each in 2013 (Anticich et al., 2013; Lee and Stewart, 2013), 2014 (Castro-Olivo, 2014; Chen et al., 2014), 2015 (Leventhal et al., 2015; Tan and Martin, 2015), 2018 (Hood et al., 2018; Yeun and Woo, 2018), and 2020 (Hatamizadeh et al., 2020; Johnstone et al., 2020). Only one study was published in 2010 (Hyun et al., 2010), 2016 (Chisholm et al., 2016), and 2017 (Li et al., 2017). Figure 2 shows the number of studies published each year.

#### Place of Study

Australia was the country where most studies were carried out (*n* = 5), followed by China (*n* = 3), United States (*n* = 2), South Korea (*n* = 2), and Iran (*n* = 2). One study was conducted in each country: Finland, India, and the United Kingdom.

#### Sample Size and Participants

Sample size ranged from 27 (Druker et al., 2019) to 2,996 (Volanen et al., 2019). Most studies included only one control group (*n* = 13). It could be an active control group that received a form of attention (Hyun et al., 2010; Tan and Martin, 2015; Chisholm et al., 2016; Hood et al., 2018; Yeun and Woo, 2018; Druker et al., 2019; Zhang et al., 2019) or an inactive control group that received no attention (Lee and Stewart, 2013; Castro-Olivo, 2014; Leventhal et al., 2015; Li et al., 2017; Adibsereshki et al., 2019; Hatamizadeh et al., 2020). Only four studies had both active and inactive control groups (Anticich et al., 2013; Chen et al., 2014; Volanen et al., 2019; Johnstone et al., 2020).

Adolescents were the target population in most studies (*n* = 11), whereas four studies had only children as participants (Anticich et al., 2013; Lee and Stewart, 2013; Druker et al., 2019; Johnstone et al., 2020) and two had children and adolescents as participants (Li et al., 2017; Zhang et al., 2019). Both females and males comprised participants sex in most studies (Anticich et al., 2013; Lee and Stewart, 2013; Castro-Olivo, 2014; Chen et al., 2014; Tan and Martin, 2015; Li et al., 2017; Hood et al., 2018; Yeun and Woo, 2018; Adibsereshki et al., 2019; Druker et al., 2019; Volanen et al., 2019; Zhang et al., 2019; Hatamizadeh et al., 2020; Johnstone et al., 2020).

#### Programs Characteristics

Each study implemented a different program. The number of sessions ranged from 5 to 23 and each session ranged from 10 to 120 min. Most programs were implemented in a school setting (*n* = 11). All programs were implemented face-to-face, and the cognitive-behavioral theory (CBT) was the most frequently approach reported (*n* = 5).

#### Measurement Tools and Follow-up

Finally, the Connor-Davidson Resilience Scale (CD-RISC) was the most used scale to assess the results (*n* = 6). Nine studies performed at least three assessments: before, after, and at least one follow-up (Anticich et al., 2013; Chen et al., 2014; Tan and Martin, 2015; Li et al., 2017; Hood et al., 2018; Adibsereshki et al., 2019; Volanen et al., 2019; Hatamizadeh et al., 2020; Johnstone et al., 2020) and eight studies had only two assessments: before and after the program (Hyun et al., 2010; Lee and Stewart, 2013; Castro-Olivo, 2014; Leventhal et al., 2015; Chisholm et al., 2016; Yeun and Woo, 2018; Druker et al., 2019; Zhang et al., 2019).

Table 1 summarizes the main characteristics of each study.

**TABLE 1:** Characteristics of included studies.

													
**References**	**Country**	***n***	**Population**	**Age**	**Sex**	**Program**	**Sessions (length)**	**Setting**	**Modality**	**Format**	**Approach**	**Scale**	**Time of assessment**
													
Adibsereshki et al. (2019)	Iran	122 (61 at EG and 61 at inactive CG)	Adolescents	12–15 years	48 females; 74 males	−	12 (75 min)	School	Group	Presential	–	Connor-Davidson Resilience Scale (CD-RISC)	3 (pre, post and 2 months follow-up)
Anticich et al. (2013)	Australia	488 (159 at EG [Fun FRIENDS], 196 at active CG [You Can Do It], and 133 at inactive CG)	Children	4–7 years	271 females; 217 males	Fun FRIENDS	12 (−)	School	Group	Presential	CBT	Devereux Early Childhood Assessment Clinical Form (DECA-C)	3 (pre, post and 12 months follow-up)
Castro-Olivo (2014)	United States	102 (49 at EG and 53 at inactive CG)	Adolescents	Mean 13.9 years	51 females; 51 males	Jovenes Fuertes − Social-Emotional Learning (SEL)	12 (−)	School	Group	Presential	–	Behavior Emotional Rating Scale (BERS-2)	2 (pre and post)
Chen et al. (2014)	China	32 (10 at EG, 10 at active CG [GS] and 12 at inactive CG)	Adolescents	Mean 14.5 years	(−) females; (−) males	Children and Disaster: Teaching Recovery Techniques	6 (60 min)	School	Group	Presential	CBT	Connor-Davidson Resilience Scale (CD-RISC)	3 (pre, post and 3 months follow-up)
Chisholm et al. (2016)	United Kingdom	657 (354 at EG and 303 at active CG [EC])	Adolescents	12–13 years	657 males	SchoolSpace	11 (10 to 60 min)	School	Group	Presential	Psychoeducational	Resilience Scale 15-item (RS-15)	2 (pre and post)
Druker et al. (2019)	Australia	27 (13 at EG and 14 at active CG [Therapy])	Children	Mean 4.3 years	11 females; 16 males	Stuttering therapy + Resilient component	12 (70 min)	Therapeutic setting	Group	Presential	–	Curtin Early Childhood Stuttering Resilience Scale (CECSRS)	2 (pre and post)
Hatamizadeh et al. (2020)	Iran	122 (61 at EG and 61 at inactive CG)	Adolescents	12 – 15 years	48 females; 74 males	–	12 (−)	School	Group	Presential	Positive psychology	Connor-Davidson resilience scale (CD-RISC)	3 (pre, post and 2 months follow-up)
Hood et al. (2018)	United States	264 (133 at EG and 131 at active CG [EC])	Adolescents	14–18 years	158 females; 106 males	Penn Resilience Program for type 1 diabetes (PRP T1D)	9 (90 to 120 min)	–	Group	Presential	CBT	Diabetes Strengths and Resilience-Teen	5 (pre, post, 4-, 8- and 12-months follow-up)
Hyun et al. (2010)	South Korea	28 (15 at EG and 13 at active CG [EC])	Adolescents	12–13 years	28 males	−	10 (50 min)	School	Group	Presential	CBT	Korean Adolescent Resilience Scale	2 (pre and post)
Johnstone et al. (2020)	Australia	232 (123 at EG [ER], 61 at active CG [BA] and 17 at inactive CG)	Children	8–12 years	(−) females; (−) males	Emotion Regulation Program (ER)/ Behavior Activation (BA)	8 (50 min)	School	Group	Presential	Emotional Regulation/Behavioral Activation	The child and youth resilience measure – short version (CYRM-12)	3 (pre, post and 6 months follow-up)
Lee and Stewart (2013)	Australia	1,277 (828 at EG and 449 at inactive CG)	Children	8–12 years	(−) females; (−) males	Health-promoting school	–	School	Group	Presential	Educational Curriculum	California Healthy Kids Survey	2 (pre and post)
Leventhal et al. (2015)	India	2,387 (1,681 at EG and 706 at inactive CG)	Adolescents	12,9 years	2,387 females	Girls First Resilience Curriculum (RC)	23 (60 min)	School	Group	Presential	Positive psychology	Connor Davidson Resilience Scale-10 (CD-RISC)	2 (pre and post)
Li et al. (2017)	China	790 (595 at EG and 195 at inactive CG)	Children and adolescents	6–17 years	382 females; 408 males	Child-Caregiver-Advocacy Resilience (ChildCARE)	15 (120 min)	–	Group	Presential	–	Connor-Davidson Resilience Scale (CD-RISC)	3 (pre, 6- and 12-months follow-up)
Tan and Martin (2015)	Australia	80 (43 at EG and 37 at active CG [TAU])	Adolescents	13–18 years	60 females; 20 males	Taming the Adolescent Mind (TAM)	5 (60 min)	Therapeutic setting	Group	Presential	Mindfulness	Resiliency Scales for Children and Adolescents (RSCA) (Prince-Embury, 2006)	3 (pre, post and 3 months follow-up)
Volanen et al. (2019)	Finland	2,996 (1,334 at EG [Stop and Breathe/Be], 1,291 at active CG [Relax] and 371 at inactive CG)	Adolescents	12–15 years	(−) females; (−) males	Stop and Breathe/Be	9 (45 min)	School	Group	Presential	–	Resilience scale 14-item (RS-14)	3 (pre, post and 4 months follow-up)
Yeun and Woo (2018)	South Korea	62 (30 at EG and 32 at active CG [TAU])	Adolescents	13–17 years	48 females; 14 males	Interpersonal Relationship improvement program (IRIP)	6 (60 min)	–	Group	Presential	–	Ego-resiliency scale	2 (pre and post)
Zhang et al. (2019)	China	106 (53 at EG and 53 at active CG [TAU])	Children and adolescents	8–18 years	54 females; 52 males	–	5 (−)	Hospital	Group	Presential	CBT	Connor-Davidson resilience scale (CD-RISC)	2 (pre and post)

*EG, experimental group; CG, control group; GS, general support; EC, educational curriculum; TAU, treatment as usual; ER, emotional regulation; BA, behavioral activation; -, Not reported; and CBT, cognitive-behavioral therapy.*

### Risk of Bias

We assessed the risk of bias according to the following domains: (D1) random sequence generation, (D2) allocation concealment, (D3) blinding of participants and personnel, (D4) blinding of outcome assessment, (D5) incomplete outcome data, (D6) selective reporting, (D7) cross contamination, and (D8) overall bias.

**FIGURE 3:**
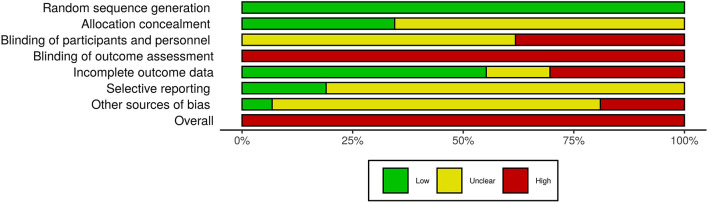
Risk of bias for all studies.

**FIGURE 4:**
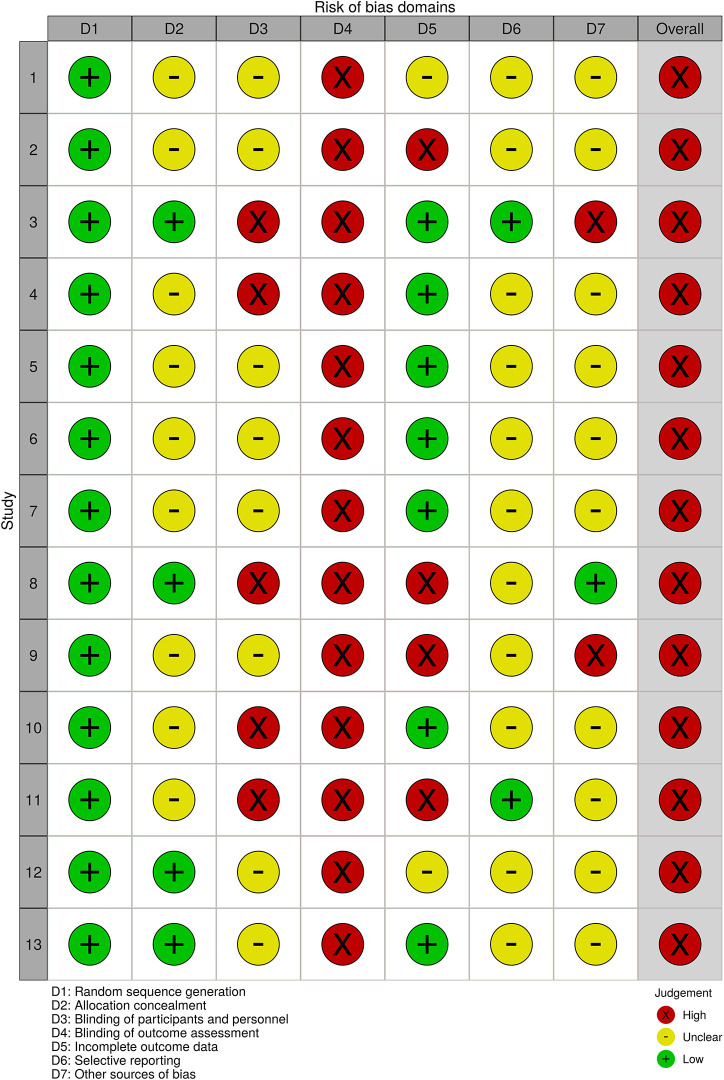
Risk of bias for each study.

As all studies were at a high risk for at least one domain, all of them were rated as having a high overall risk of bias (D8). All studies were rated as a low risk for (D1) random sequence generation, as we only included RCTs in the systematic review. As the aims of the studies were to assess the programs’ efficacy, it was difficult to ensure the blinding of participants and personnel, and the outcome of every study was assessed through self-report measures; therefore, the blinding of outcome could not be ensured as well. Therefore, these domains (D3 and D4) were frequently rated as unclear or a high risk. Similarly, a few studies could ensure that cross contamination (D7) between participants of different groups did not occur. Figure 3 shows the risk of bias for each domain for all studies and Figure 4, for each domain for each study.

**FIGURE 5:**
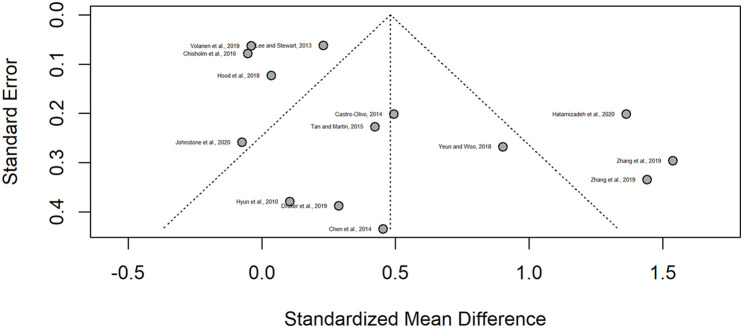
Publication risk funnel plot.

Publication bias was assessed visually through a funnel plot inspection (Figure 5), which considered the SMD and SE of the studies. The funnel plot did not indicate considerable asymmetry, as the intersection on the *x*-axis was close to zero. Egger test performed for asymmetry confirmed this result (*p* = 0.02). Therefore, the likelihood of publication bias could be considered low.

### Quantitative Results

Of the 17 studies included in this review, four did not provide the necessary statistical data for their inclusion in the meta-analysis (Anticich et al., 2013; Leventhal et al., 2015; Li et al., 2017; Adibsereshki et al., 2019). We contacted the authors but did not obtain a reply. Consequently, only 13 studies were included in the meta-analysis (Hyun et al., 2010; Lee and Stewart, 2013; Castro-Olivo, 2014; Chen et al., 2014; Tan and Martin, 2015; Chisholm et al., 2016; Hood et al., 2018; Yeun and Woo, 2018; Druker et al., 2019; Volanen et al., 2019; Zhang et al., 2019; Hatamizadeh et al., 2020; Johnstone et al., 2020).

For studies that had multiple control groups (active and inactive control groups), we opted to conduct the analyses with the inactive control groups to compare if the interventions would be better than no treatment. The study of Johnstone et al. (2020) included two experimental groups: Emotional Regulation (ER) and Behavioral Activation (BA); but for the meta-analysis, we considered the ER group, as the main objective of their study was to assess the effectiveness of a novel treatment. In addition, Zhang et al. (2019) analyzed their results in subgroups, a sample of children, and another sample of adolescents; therefore, this study had two different samples for control and experimental groups, and it was considered twice in our analysis.

The random SMD of meta-analysis indicated an overall increase in resilience immediately after the completion of programs, thereby supporting the intervention (SMD = 0.48, 95% CI [0.15, 0.81], *p* = 0.0077). Heterogeneity among studies might be considered high (*I*^2^ = 88%, 95% CI [81%, 92%], *p* < 0.001).

### Subgroup Analysis

Subgroup analyses were also performed. The first subgroup comprised adolescents (12–22 years old). Ten studies provided data for this analysis (Hyun et al., 2010; Castro-Olivo, 2014; Chen et al., 2014; Tan and Martin, 2015; Chisholm et al., 2016; Hood et al., 2018; Yeun and Woo, 2018; Volanen et al., 2019; Zhang et al., 2019; Hatamizadeh et al., 2020). Results for this subgroup analysis indicated a significant increase in resilience (SMD = 0.48, 95% CI [0.08, 0.88], *p* = 0.02). Heterogeneity among studies was also high (*I*^2^ = 89%, 95% CI [81%, 93%], *p* < 0.001).

The second subgroup was composed exclusively of children (<12 years old). Four studies provided enough data for this analysis (Lee and Stewart, 2013; Druker et al., 2019; Zhang et al., 2019; Johnstone et al., 2020). The results of this subgroup indicated no significant increase in resilience (SMD = 0.48, 95% CI [−0.64, 1.61], *p* = 0.26). Although only four studies were included, the heterogeneity of the sample could be considered high (*I*^2^ = 85%, 95% CI [64%, 94%], *p* < 0.001).

**FIGURE 6:**
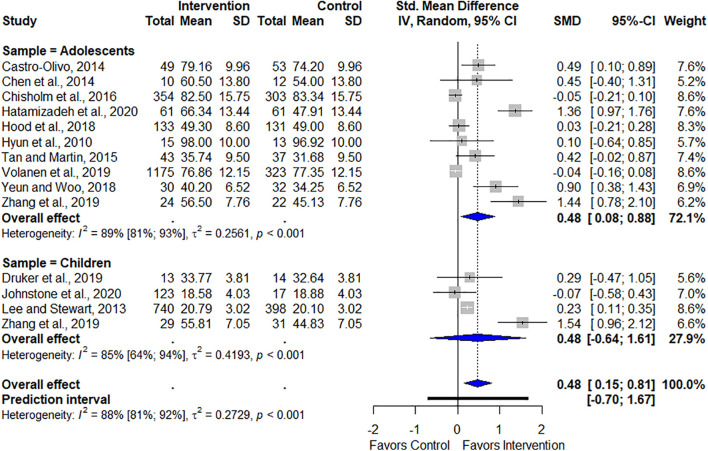
Overall and subgroup effects of programs on resilience.

Figure 6 graphically represents the overall and subgroup results of the programs’ effectiveness in promoting resilience immediately after their conclusion.

### Follow-up Analysis

Four studies conducted short-term follow-up (≤3 months) (Chen et al., 2014; Tan and Martin, 2015; Adibsereshki et al., 2019; Hatamizadeh et al., 2020), but two of which did not provide the necessary data to be included in this analysis (Anticich et al., 2013; Adibsereshki et al., 2019). Therefore, only three studies were included in the short-term follow-up period. This analysis did not indicate significant results in the short-term (SMD = 0.96, 95% CI [0.14, 1.78], *p* = 0.26). Its heterogeneity was considered moderate (*I*^2^ = 46%, 95% CI [0%, 84%], *p* = 0.15).

Four studies conducted mid-term follow-up (3 to 6 months) (Li et al., 2017; Hood et al., 2018; Volanen et al., 2019; Johnstone et al., 2020), but one study could not be included because the authors did not provide the results of the follow-up (Li et al., 2017). Hood et al. (2018) and Volanen et al. (2019) performed a follow-up at 4 months, whereas Johnstone et al. (2020) performed only one follow-up at 6 months. This analysis indicated continuation of results in the mid-term follow-up (SMD = 0.12, 95% CI [−0.44, 0.69], *p* = 0.02). Heterogeneity was considered moderate (*I* = 39%, 95% CI [0%, 81%], *p* = 0.19).

Three RCTs conducted long-term follow-up (> 6 months; Anticich et al., 2013; Li et al., 2017; Hood et al., 2018). Although Anticich et al. (2013) and Li et al. (2017) also performed a 12-month follow-up, they provided insufficient data. Hood et al. (2018) reported two long-term follow-ups (8 and 12 months); we opted to use the 12-month follow-up, as it was the longest assessment.

**FIGURE 7:**
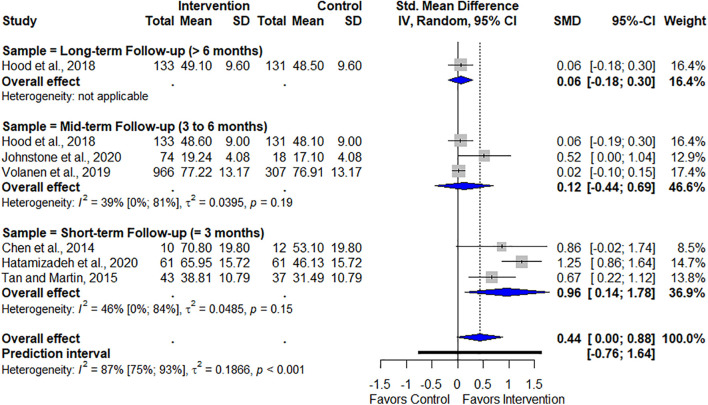
Follow-up effects of program on resilience.

The overall follow-up analysis indicated the programs’ effectiveness in maintaining an enhanced resilience, thereby supporting the intervention [SMD = 0.44, 95% CI [0.00, 0.88], *p* = 0.05). Heterogeneity was considered high (*I*^2^ = 87%, 95% CI [75%, 93%], *p* < 0.001). Figure 7 graphically shows the follow-up results.

## Discussion

This systematic review aimed to address the effectiveness of resilience programs for children and adolescents. To our knowledge, this is the first systematic review with this aim. Our main findings indicate that such programs are effective in promoting overall resilience. The subgroup analysis in the present review did not indicate changes in resilience for children, but did for the adolescents’ subgroup, indicating significant results in enhancing resilience for this population. These findings may be due to the reduced number of studies in the children’s subgroup, which may not have been enough to provide significant changes in resilience levels. Although childhood and adolescence are characterized by brain development and the acquisition of important cognitive functions, the adolescent phase has a greater tendency to associate with peers (Masten and Barnes, 2018). This tendency of association may favor the expansion of social support networks and work as an extra facilitator that contributes to greater resilience alongside the implementation of resilience-focused programs.

Our follow-up analysis showed that results are maintained for up to six months. In this direction, other studies demonstrate that results of these programs also seem to be maintained for months after the end of programs (Leppin et al., 2014; Sander et al., 2016; Dray et al., 2017). However, differently from these findings, Vanhove et al. (2015) did not verify the maintenance of resilience-focused programs in enhanced emotional well-being in a follow-up analysis, but unlike the present study, Vanhove et al.’s (2015) review included only studies that implemented programs in working settings and included only the adult population, which may have contributed to such findings.

Resilience has become an increasingly popular topic, and programs focused on its promotion have been developed and studied in the last years. As some reviews show, resilience programs may have a diverse range of characteristics, such as different populations, theoretical approaches, quantity and length of sessions, and settings of implementation (Dray et al., 2017; Helmreich et al., 2017; Laird et al., 2019). Analyzing these aspects of programs, most RCT included in our systematic review implemented programs with CBT approaches. In the same direction, CBT was one of the most frequently approaches identified by other systematic reviews (Leppin et al., 2014; Dray et al., 2017). Our study identified that the programs ranged from 5 to 23 sessions. Similarly, Dray et al. (2017) report that the programs analyzed by their systematic review ranged from 5 to 32 weeks, Leppin et al. (2014) report programs ranging from 1 to 24 sessions, and Hodder et al. (2017) identified programs lasting from 2 days to 10 years. Each session of the programs included in the present review ranged from 10 to 120 min. Dray et al.’s (2017) systematic review indicate similar results, with sessions ranging from 15 to 120 min, whereas Leppin et al. (2014) report longer sessions ranging from 40 to 150 min. In the present review, adolescents were the target population in majority of the included RCT and face-to-face programs implemented at the school were the most frequent. The school setting was also one of the most frequently reported by other systematic reviews when children and/or adolescents were the target population (Dray et al., 2017; Hodder et al., 2017; Fenwick-Smith et al., 2018). Additionally, self-report scales were frequently used among the included studies, and CD-RISC was the most reported measurement tool to assess resilience. Although other systematic reviews have analyzed the effects of programs on different outcomes, self-report scales were also the most used measurement tool to assess the effectiveness of such programs (Sander et al., 2016; Dray et al., 2017).

Despite this diversity of characteristics, such programs may lead to diverse beneficial outcomes, not only improving resilience itself, but also decreasing stress and depression (Leppin et al., 2014), anxiety symptoms and psychological distress, internalizing problems (Dray et al., 2017), and reducing consumption of illicit substances (Hodder et al., 2017).

The results of the present study indicated the effectiveness of programs with psychotherapeutic strategies in promoting resilience but although the promising results, these findings should be interpreted with caution. Some limitations of this study must be considered. The first limitation refers to the impossibility of including four studies in the main meta-analysis because of the lack of available data, and most of the included studies were rated as having a high overall risk of bias. This high risk of bias, however, seems to be a common result in systematic reviews of resilience programs; it could even be an expected result, as other systematic reviews that also assessed the risk of bias of mental health and psychological programs had similar conclusions (Dray et al., 2017; Hodder et al., 2017). The second limitation refers to the variation in the sample size of studies, different number of sessions, diverse program approaches, and diversity of scales used to assess the outcomes. This diversity in characteristics across studies might lead to diverse results, which makes drawing definitive conclusions on programs efficacy more difficult. Finally, the heterogeneity of studies might be considered high, even in the subgroup analysis. However, this heterogeneity could also be expected, given the diversity of the studies and the fact that similar reviews found similar results, ranging from moderate (Vanhove et al., 2015; Hodder et al., 2017; Joyce et al., 2018) to high heterogeneity (Dray et al., 2017; Joyce et al., 2018).

Still, this systematic review provides an overview of existing resilience-focused programs for children and adolescents and provides relevant data for the field, as our findings may help to guide future actions and interventions aimed to promote resilience. By implementing such interventions as early as possible with the juvenile population, we may promote not only resilience and our ability to cope and recover from the adversities that are so common in today’s world, but also promote better public health outcomes as more resilient individuals tend deal better with situations of adversity that can facilitate mental health problems. Therefore, the school may be a key setting for carrying out such programs, as we find many children and adolescents gathered in schools.

## Conclusion

We might conclude that the present systematic review contributes to the body of evidence in the field of resilience programs, as it provides an overview of resilience-focused programs for children and adolescents and our results suggest its effectiveness in promoting resilience, especially among adolescents. Additionally, these results are maintained for up to six months as shown in follow-up analysis.

It is noteworthy that future studies that analyze the effectiveness of programs with different characteristics from those included in the present review, such as interventions implemented online or individually, are still necessary to contribute to the growing evidence in this field and to help developing increasingly effective interventions.

## Data Availability Statement

The raw data supporting the conclusions of this article will be made available by the authors, without undue reservation.

## Author Contributions

TP and EM designed the study. TP and PL performed the search and data extraction. PL performed the statistical calculations. TP wrote the first draft of this manuscript. TP, CM, PL, and EM participated in the review and contributed to the writing. All authors contributed to the article and approved the final version of the manuscript.

## Conflict of Interest

The authors declare that the research was conducted in the absence of any commercial or financial relationships that could be construed as a potential conflict of interest.

## Publisher’s Note

All claims expressed in this article are solely those of the authors and do not necessarily represent those of their affiliated organizations, or those of the publisher, the editors and the reviewers. Any product that may be evaluated in this article, or claim that may be made by its manufacturer, is not guaranteed or endorsed by the publisher.

## Appendix

### Appendix 1. Search strategy

(((resilien^∗^ NEAR/5 (train^∗^ OR program^∗^ OR intervention^∗^ OR promot^∗^ OR prevent^∗^ OR enhanc^∗^ OR learn^∗^ OR teach^∗^ OR educat^∗^ OR increas^∗^ OR develop^∗^ OR manag^∗^ OR therap^∗^ OR protocol^∗^ OR treat^∗^)) OR (hardiness^∗^ NEAR/5 (train^∗^ OR program^∗^ OR intervention^∗^ OR promot^∗^ OR prevent^∗^ OR enhanc^∗^ OR learn^∗^ OR teach^∗^ OR educat^∗^ OR increas^∗^ OR develop^∗^ OR manag^∗^ OR therap^∗^ OR protocol^∗^ OR treat^∗^)) OR ((‘psychological resilience’/exp OR ‘social adaptation’/exp OR ‘coping behavior’/exp OR ‘post-traumatic growth’ OR ‘posttraumatic growth’ OR ‘stress-related growth’ OR (positiv^∗^ NEAR/1 (adapt^∗^ OR adjust^∗^)) OR resilien^∗^ OR hardiness^∗^ OR cope OR coping OR ((overcom^∗^ OR resis^∗^ OR recover^∗^ OR thri^∗^ OR adapt^∗^ OR adjust^∗^) NEAR/5 (stress^∗^ OR trauma^∗^ OR adversit^∗^)) OR ‘psychologic adaptation’ OR ‘psychological adaptation’) AND (‘psychotherapy’/exp OR ‘psychological stress’ OR ‘psychological stresses’ OR psychotherap^∗^ OR psycho-therap^∗^ OR (behav^∗^ NEAR/3 (intervention^∗^ OR program^∗^ OR therap^∗^)) OR ((cbt OR ‘cognitive behavioral’ OR ‘cognitive behavior’ OR cognition) NEAR/3 (intervention^∗^ OR program^∗^ OR therap^∗^)) OR (psycho^∗^ NEAR/3 (intervention^∗^ OR program^∗^ OR therap^∗^)) OR counseling OR coaching OR mindful^∗^ OR relaxation OR (‘third wave’ NEAR/1 (psycho^∗^ OR therap^∗^)) OR ‘cognitive restructuring’ OR ‘positive psychology’ OR refram^∗^ OR re-fram^∗^ OR reapprais^∗^ OR (stress NEAR/1 (inoculation OR manag^∗^ OR reduc^∗^ OR resist^∗^)) OR (anxiety NEAR/3 manage^∗^) OR ‘acceptance and commitment’ OR ‘health promotion’/exp OR (health NEAR/3 (educat^∗^ OR promot^∗^))))) AND (‘randomized controlled trial’/exp OR ‘single blind procedure’/exp OR ‘double blind procedure’/exp OR ‘crossover procedure’/exp OR random^∗^:ab,ti OR placebo^∗^:ab,ti OR allocat^∗^:ab,ti OR crossover^∗^:ab,ti OR ‘cross over’:ab,ti OR trial:ti OR ((doubl^∗^ NEXT/1 blind^∗^):ab,ti)) NOT ((‘animal’/de OR ‘animal experiment’/de OR ‘nonhuman’/de) NOT ((‘animal’/de OR ‘animal experiment’/de OR ‘nonhuman’/de) AND ‘human’/de)) AND (1990:py OR 1991:py OR 1992:py OR 1993:py OR 1994:py OR 1995:py OR 1996:py OR 1997:py OR 1998:py OR 1999:py OR 2000:py OR 2001:py OR 2002:py OR 2003:py OR 2004:py OR 2005:py OR 2006:py OR 2007:py OR 2008:py OR 2009:py OR 2010:py OR 2011:py OR 2012:py OR 2013:py OR 2014:py OR 2015:py OR 2016:py OR 2017:py OR 2018:py OR 2019:py OR 2020:py OR 2021:py) AND ([adolescent]/lim OR [child]/lim OR [preschool]/lim OR [school]/lim OR [young adult]/lim)) AND (2020:py OR 2021:py)
